# Evaluation of 10-Nitro Oleic Acid Bio-Elimination in Rats and Humans

**DOI:** 10.1038/srep39900

**Published:** 2017-01-05

**Authors:** Sonia R. Salvatore, Dario A. Vitturi, Marco Fazzari, Diane K. Jorkasky, Francisco J. Schopfer

**Affiliations:** 1 Department of Pharmacology and Chemical Biology, University of Pittsburgh, Pittsburgh, PA, 15261, USA; 2 Fondazione Ri.MED, Palermo, 90133, Italy; 3 Complexa Inc., Pittsburgh, PA, 15203, USA

## Abstract

Nitrated fatty acids are endogenously present in human and animal tissues, as well as in plant-derived oils. In particular, 10-nitro oleic acid (10-NO_2_-OA) potently induces Nrf2-dependent antioxidant gene expression and inhibits TLR4/NF-κB signaling, thus promoting an overall cyto-protective and anti-inflammatory response. 10-NO_2_-OA has been extensively tested in animal models and is currently undergoing clinical evaluation in humans. Bio-elimination pathways for 10-NO_2_-OA were evaluated in rats (30 mg/kg·day) and in humans (0.34 mg/kg) using samples obtained from a double-blind, dose-rising clinical trial. Quantitative radiochromatographic/MS analysis indicated that the renal and fecal pathways are the main routes for 10-NO_2_-OA excretion in rats, and allowed the identification of 4-nitro-octanedioic acid (NO_2_-8:0-diCOOH) as the most abundant metabolite in rat urine. In addition, high resolution LC-MS/MS analysis revealed the presence of a novel series of urinary metabolites including ω-carboxylation and β-oxidation products, as well as N-acetylcysteine, taurine and sulfo-conjugates in both rats and humans. Overall, the findings reported herein not only provide valuable tools for the experimental evaluation of 10-NO_2_-OA levels *in vivo*, but importantly they also set the basis for monitoring its metabolism during potential clinical interventions in humans.

Nitrated fatty acids are formed during digestion and inflammatory processes from the reaction between nitrogen dioxide (^•^NO_2_) and mono and polyunsaturated fatty acids in both humans and animal models^1^,^2^,^3^,^4^. In particular, nitric oxide (^•^NO) autoxidation, nitrous acid disproportionation, enzymatic oxidation of nitrite (NO_2_^−^), and peroxynitrite homolysis lead to ^•^NO_2_ formation and fatty acid nitration *in vitro* and *in vivo*^1^,^3^,^5^,^6^. The nitroalkene moiety in nitrated fatty acids conveys electrophilic properties to these molecules promoting Michael addition reactions with cysteine and histidine residues^7^,^8^. Nitrated fatty acids are detected in mouse heart tissue after ischemia reperfusion, are endogenously present in humans and their levels can be modulated by dietary interventions^1^,^2^,^9^,^10^,^11^,^12^.

Nitro oleic acid (NO_2_-OA) is a component of the nitrated fatty acid pool in human and animal tissues, and is also present in plant-derived oils^13^,^14^,^15^. The endogenous levels of the positional isomers 9- and 10-NO_2_-OA in humans are 0.3–1 nM each in the circulation^14^,^15^, and 5 fmoles/mg creatinine of total NO_2_-OA in urine^16^. The discovery of the modulatory effects of NO_2_-OA on cyto-protective and inflammatory signaling pathways (*e*.*g*. activation of Nrf2- and heat shock responses, TLR4/NF-κB inhibition) led to its evaluation in animal models of disease and more recently to its pre-clinical development for use in humans^17^,^18^,^19^,^20^. Oral, subcutaneous or intravenous administration of NO_2_-OA is protective in animal models of inflammatory bowel disease, heart and kidney ischemia reperfusion, adriamycin-induced renal toxicity, restenosis after endoluminal injury, lung injury, atherosclerosis development, endotoxemia and diabetes among others^10^,^21^,^22^,^23^,^24^,^25^,^26^,^27^,^28^,^29^. An initial characterization of NO_2_-OA metabolism in mice revealed the presence of secondary nitrated species that included β-oxidation products, non-electrophilic nitroalkene reduction derivatives, glutathione conjugates and coenzyme A esters^30^. Furthermore, the incorporation of NO_2_-OA metabolites into triglycerides was recently reported in rodent adipocytes^31^. Notably, whereas published studies described NO_2_-OA metabolism following intravenous infusion, little is known about bioavailability, biodistribution and excretion of these species upon oral administration^1^. Moreover, the metabolic profile of NO_2_-OA in humans is completely unknown.

This report defines pathways and metabolic routes involved in 10-NO_2_-OA bio-elimination in rats using [^14^C]-10-NO_2_-OA radiochromatographic techniques, and identifies a novel series of urinary metabolites including ω-carboxylation and β-oxidation products, as well as N-acetylcysteine-, taurine- and sulfo-conjugates in both rats and humans via mass spectrometry analysis. These findings not only provide critical tools for future evaluation of endogenous nitro-fatty acid formation *in vivo*, but also establish a solid framework to reliably monitor NO_2_-OA metabolism within the context of human pharmacological interventions.

## Materials and Methods

10-Nitro-oleic acid (10-NO_2_-OA) was synthesized as previously^32^. 10-[^15^N]O_2_-d_4_-OA ((E)-10-(nitro-^15^N) octadec-9-enoic-15,15,16,16-d4 acid) was synthesized as 10-NO_2_-OA but using 1-Bromononane-6,6,7,7-*d*_*4*_ and Na[^15^N]O_2_ as starting materials). Chemical and isotopic purities of 10-[^15^N]O_2_-d_4_-OA were ≥95% and ≥99% respectively as determined by NMR and LC-MS analyses. Chemicals were purchased from Sigma (St. Louis, MO) unless otherwise indicated. [^14^C]-10-NO_2_-OA (labeled on carbon 10), specific activity 0.885 MBq/mg (7.83 mCi/mmol), was synthesized by ABC Laboratories, Inc., showing a radiochemical purity of 99.7%, as determined by HPLC with on-line radioactivity detection.

### Ethics statement

#### Animal Studies

Animals were housed in accordance with the Guide for the Care and Use of Laboratory Animals published by the United States National Institutes of Health (NIH Publication No. 85-23, revised 1996). All rodent and clinical studies were approved by the University of Pittsburgh Institutional Animal Care and Use Committee (Approval 15096581). Radiolabeling studies were performed at Huntingdon Life Sciences laboratories (Huntingdon, United Kingdom) in compliance with the relevant guidelines issued by the UK Home Office, from whom Project and Personal Licenses were obtained as specified by the Animals (Scientific Procedures) Act 1986 following the European Union Directive 86/609/EEC which requires ethics review of the Licenses and their Amendments and approval by the HLS Ethical Review Process Committee.

#### Clinical Trial

All subjects recruited for the randomized trial (ClinicalTrials.gov # NCT02127190, registered on March 24^th^, 2014) provided written informed consent and ethics oversight was provided by the IRB#PROCXA-10-001 and the University of Pittsburgh Human Subjects IRB #PRO15070300.

All methods were carried out in accordance with relevant guidelines and regulations. All experimental protocols were approved by the University of Pittsburgh Institutional Animal Care and Use Committee (Approval 15096581), the University of Pittsburgh Human Subjects Institutional Review Board (#PRO15070300), Complexa (IRB#PROCXA-10-001) and the HLS Ethical Review Process Committee. Informed consent was obtained from all subjects.

### Clinical trial design

The clinical trial reported herein is a sub-study of a larger randomized, double-blind, dose-rising study of 10-NO_2_-OA in healthy volunteers. Healthy male and female volunteers ages 18 to 50 years with a body mass index (BMI) between 18 and 30 kg/m^2^ (inclusive) and a weight between 60 kg and 100 kg (inclusive) were recruited from the general population in Kalamazoo, MI. Subjects (n = 9) were randomized to three cohorts that received a 60 min intravenous administration of placebo (n = 3) or emulsified 10-NO_2_-OA (10 mg 10-NO_2_-OA/ml in a formulation containing soybean oil, medium chain triglycerides oil, egg phospholipids, sucrose, and disodium EDTA)(n = 6) at each dose levels. This dose escalation study consisted of the following doses: 0.01, 0.015, 0.043, 0.12, 0.34 and 0.68 mg/kg (equivalent to 0.38, 0.57, 1.63, 4.7, 13 and 26 mg/m^2^). For this sub-study, urine samples from subjects receiving 0.34 mg/kg dose (13 mg/m^2^) were used. Urine collected during the first 12 hours was analyzed. A washout of 15 days was established between the dose corresponding to 0.1 mg/kg and the dose analyzed in this study. Washout times between the other dose levels were between 14 and 19 days. The sample size for this sub-study was determined based on the availability of resources to conduct the specialized laboratory measures and the current report describes the trial’s secondary outcomes related to the clinical identification of 10-NO_2_-OA metabolites.

#### Inclusion criteria

Individuals in good general health as determined by a thorough medical history and physical examination, ECG, vital signs, and clinical laboratory evaluation with clinical laboratory tests without clinically significant abnormalities, including hematology, clinical chemistry and urinalysis. Subjects with resting heart rates (HR) ≥50 beats per minute at baseline with QTcF interval (Fredericia’s correction factor) of the baseline ECG between ≤430 ms for males and ≤450 ms for females at screening and predose and adequate bilateral venous access to allow for repeated dose infusions and blood sampling.

#### Exclusion Criteria

Subjects with any other clinically relevant ECG parameter abnormality (e.g., PR interval, QRS deviation) or any clinically significant ECG abnormality including a history of congenital long QT syndrome in the subject or in the subject’s family. Female subject pregnant or lactating, or with positive urine β-human chorionic gonadotropin (β-hCG) test. Volunteers with any clinical history of cardiovascular events, arrhythmias, fainting, palpitations, personal or family history of congenital prolonged QT syndromes, history of any primary malignancy, including a history of melanoma or suspicious undiagnosed skin lesions, with the exception of basal cell or squamous cell carcinomas of the skin or cervical carcinoma *in situ* or other malignancies curatively treated and with no evidence of disease for at least 5 years. Subjects with history of pancreatitis, hypersensitivity to eggs or soy products, history of regular alcohol consumption (exceeding 14 units/week for women or 21 units/week for men), smoking or use of nicotine products, high energy drinks, smoking cessation products, any prescription or non-prescription. Subjects with sitting blood pressure >140 mmHg systolic and/or >90 mmHg diastolic after 5 minutes rest (feet on floor, arm held at level of heart), PR interval >200 ms or <120 ms, abnormalities on 12-lead ECG at the screening visit for subjects, or any other cardiovascular abnormality including Pathological Q-waves, murmur and ventricular pre-excitation.

#### Rat dosing and sample collection

Six male Sprague-Dawley rats, were housed in solid-bottom polycarbonate cages and acclimatized for two weeks and then transferred into polycarbonate metabolic cages. Then the rats, weighing 211–226 g, were gavaged for two days with a daily dose of 30 mg/kg 10-NO_2_-OA dissolved in 200 μl sesame oil. Urine was collected at pre-dose and at 6 h post-gavage, protected from light and stored at −80 °C until use (within a month of collection). All data and chromatograms shown correspond to samples obtained during the first 6 h collection.

#### Rat radiolabeling excretion studies

Oral doses of [^14^C]-10-NO_2_-OA (labeled at carbon 10) were administered to 4 Sprague-Dawley CD male rats weighing 234–272 g by gastric intubation at a dose level of 30 mg/4 MBq/2 ml sesame oil/kg body weight. The animals were housed singly in glass metabolic cages to allow collection of urine and feces (at pre-dose, 6 h and subsequently every 24 h post administration) into light protected containers cooled in solid CO_2_. Radioactivity present as [^14^C]O_2_ in exhaled air was collected in 1 M potassium hydroxide and cage interiors were thoroughly washed with water every 24 hours, and a last wash with methanol was performed at the end of the seven-day study. Carcasses were digested for 24 hours at 55 °C in sodium hydroxide 2 M plus 10% Triton X-100 in 30% methanol in water. Radioactivity was measured by liquid scintillation analysis using Ultima Gold scintillation cocktail and a Wallac 1409 automatic liquid scintillation counter. For metabolite identification studies, pooled urine samples were extracted using C18 solid-phase cartridges preconditioned with acetonitrile (5 ml) and water (5 ml). The cartridge was then washed with 0.1% aqueous formic acid (2 ml) and the radioactivity eluted with 5% acetonitrile (2 ml) followed by 100% acetonitrile (2 ml). Samples were chromatographically resolved using a C18 column (YMC Pack ODS-AQ, 150 × 4.6 mm id; 3 μm) at a 1 ml/minute flow using 0.1% formic acid in water (A) and 0.1% formic acid in acetonitrile (B) as the mobile phase. Samples were loaded at 3% B maintaining solvent composition for 10 minutes, followed by an increase to 25% B over 20 minutes and a final increase to 90% B over the next 30 minutes. The column was then washed for 5 minutes and re-equilibrated for 18 minutes at 3% B. The eluate was split in a ratio of ca 8:2, with the majority of the flow to the fraction collector (fractions collected at 0.25 minutes/well) for radioactivity measurement and the remainder diverted to the mass spectrometer.

#### Sample processing for human and rat metabolite profiling

Urine samples from rats (2 mL) and humans (5 mL) were thawed at room temperature, spiked with 10-[^15^N]O_2_-d_4_-OA (1.66 μg/ml for rats and 0.66 μg/ml for humans) acidified with 1% of formic acid and loaded onto 500 mg C18 Sep-Pak (ThermoFisher Scientific) pre-equilibrated with H_2_O containing 5% methanol and 0.1% formic acid. The column was washed with 4 volumes of pre equilibration solution, dried and metabolites eluted with 1 ml of 100% methanol containing 0.1% formic acid. Solvent was evaporated and samples reconstituted in 150 ml of methanol. 10-[^15^N]O_2_-d_4_-OA was utilized as an internal standard to perform 10-NO_2_-OA quantification in urine, control for extraction efficiency and as a reference standard to establish relative retention times and to accurately compare HPLC-MS/MS chromatograms between runs and across species.

#### HPLC-MS/MS Analysis

The extracts containing the metabolites were analyzed by HPLC-ESI MS/MS using a gradient solvent systems consisting of H_2_O containing 0.1% acetic acid (solvent A) and ACN (acetonitrile) containing 0.1% acetic acid (solvent B). Metabolites were resolved using a reverse phase HPLC column (100 × 2 mm id; 5 μm particle size C18 Luna column; Phenomenex) at a 0.65 ml/min flow rate. Samples were loaded at 6% B and after 1 minute a linear gradient was developed over the following 19 min to reach 100%B. Metabolites were identified and further characterized using an LTQ Velos Orbitrap (Velos Orbitrap, ThermoScientific) equipped with a HESI II electrospray source in both CID and HCD modes and in an API 5000 triple quadrupole mass spectrometer (Applied Biosystems; San Jose, CA) equipped with an electrospray ionization source. A triple quadrupole mass spectrometer was used to search for precursors of *m*/*z* 46 (in negative ion mode) to screen for metabolites containing a NO_2_ group, as this is a sensitive and well characterized method to identify NO_2_-containing ions^32^,^33^. Full MS/MS data was obtained on peaks revealing a fragment ion with *m*/*z* 46 upon CID fragmentation and levels of putative metabolites compared before and after 10-NO_2_-OA oral administration. Samples were evaluated in parallel using a hybrid high resolution Velos Orbitrap. Initially, full MS scans were obtained between 100–650 *m*/*z* and MS/MS data was obtained for the 5 most intense peaks. Differential analysis was used to determine the presence of metabolites derived from 10-NO_2_-OA both in negative and positive ion mode that might not have contained a NO_2_ and thus remained silent upon triple quadrupole targeted analysis. The following parameters for the mass spectrometers were used: 1) Orbitrap Velos, source temperature 500 °C, capillary temperature 130 °C, sheath gas flow 24, auxiliary gas flow 20, sweep gas flow 20, source voltage - 5 kV, S-lens RF level 69 (%) and 2) API 5000, Electrospray voltage was –4.5 kV, declustering potential −50 eV, CID −35, gas1 55 and gas2 60 and the source temperature was set at 600 °C.

#### Composition analysis

Atomic composition analysis was performed on high resolution MS data at the 5 ppm level to establish the number of carbon, nitrogen, hydrogen, oxygen and sulfur atoms present in each metabolite. Potential contribution of non-covalent adducts was evaluated by manual inspection of MS data, co-elution and mass analysis.

## Results

### Excretion balance

To determine the contribution of the various 10-NO_2_-OA routes of elimination, excreted radioactivity was measured over four days following a single dose of [^14^C] 10-NO_2_-OA. Urinary excretion accounted for 34.9% of the total radioactivity recovered, with an 89% of the cumulative total amount measured in the first 24 h post dose and increasing to 96% during the initial 48 h. Fecal content of radioactivity amounted to 47.9% with 98% excreted during the first 48 h. Radioactivity measured in fecal samples accounts for both non-absorbed product and its metabolites, as well as for bile-disposed products. Only 2% was excreted through exhaled air, 4.5% remained in the carcasses and 2% was retrieved from the cage washes.

### Radiochromatographic detection of major metabolites in urine

The use of [^14^C]-10-NO_2_-OA as a radiolabeled probe in combination with HLPC separation of the resulting urine extracts, provides accurate quantitative information on the relative abundance of individual 10-NO_2_-OA metabolites, for which no isotopically labeled internal standards are available. Urine collected during the excretion balance studies was subjected to simultaneous radiochromatographic/MS analysis to identify the most prominent NO_2_-OA elimination metabolites. The main radioactive component representing 30.2% of urine radioactivity in the 6 h sample, 20.9% at 6–24 h and 7.81% of the total radioactivity administered, displayed a *m*/*z* of 242 in positive ion mode which corresponded to a [M + Na]^+^ adduct and a *m*/*z* 218 in negative ion mode [M − H]^−^. This product was identified as metabolite C5 by high resolution LC-MS/MS analysis (*vide infra*). A second metabolite representing 23.1% of the radioactive signal in the first 6 h and 19.4% in the 6–24 h period (6.68% of the total dose) had an apparent mass of *m*/*z* 211 in positive ion mode corresponding to a [M + Na]^+^ and *m*/*z* 187 [M − H]^−^ in negative ion mode. The displayed mass was consistent with a dicarboxylic acid containing a keto group, although we were unable to confirm its elemental composition by high resolution HPLC-MS/MS analysis. Finally, a third major metabolite was identified comprising 10% of the radioactive signal in the 0–6 h window and 14% during the 6–24 h period (3.78% total dose). This metabolite displayed a *m*/*z* 349 [M + Na]^+^ and *m*/*z* 325 [M − H]^−^ in positive and negative ion modes respectively, consistent with it being an 8 carbon-long dicarboxylic taurine conjugate, later identified as metabolite F3. Overall, these three metabolites accounted for 18.3% of total radioactive material and 52.4% of total urinary metabolites. Additional peaks each contributing to less than 2% of the total dose were detected but proved difficult to characterize.

### Metabolite profiling

To generate a more inclusive metabolic profile of 10-NO_2_-OA, covering less abundant metabolites and to establish the routes involved in its disposal, additional targeted and untargeted LC-MS/MS analyses were performed in rat urine. In addition, human urine samples from a NO_2_-OA clinical trial were analyzed to determine whether rat metabolites are conserved across species and to define the presence of human-specific metabolites. Putative 10-NO_2_-OA metabolites were identified by paired evaluation of LC-MS/MS profiles derived from a targeted approach monitoring collision-induced NO_2_^−^ ion formation, in conjunction with untargeted high resolution MS analysis. This approach resulted in a set of ions of interest defined by particular MRMs. The specific formation of these ions was confirmed by scanning both pre-dose and after 10-NO_2_-OA dose samples in negative ion mode. All ions that met the criteria of not being present in pre-dose samples but detectable in post-dose analysis were further characterized by high resolution mass spectrometry when permitted by the sensitivity of the mass spectrometer responses. The parent 10-NO_2_-OA was detected at low levels in both rat and human urine with a retention time of 16.22 min and co-eluting with the deuterated 10-[^15^N]O_2_-d_4_-OA internal standard by following the corresponding MRM 326.2/46 and 331/47 transitions. Measured urinary levels of 10-NO_2_-OA after supplementation were 8.40 ± 3.24 nM (0–12 h collected urine) and 66.88 ± 29.91 nM (0–6 h collected urine) in humans and rats respectively. All other 10-NO_2_-OA derivatives are presented below classified according to the metabolic pathways involved in their generation. Basal levels of endogenous 9- or 10-NO_2_-OA were not detected in either rat or human urine. Nonetheless, the presence of NO_2_-12:1 was detected in low amounts in three out of six human urine samples.

### Beta-oxidation metabolite group A

**Figure 1:**
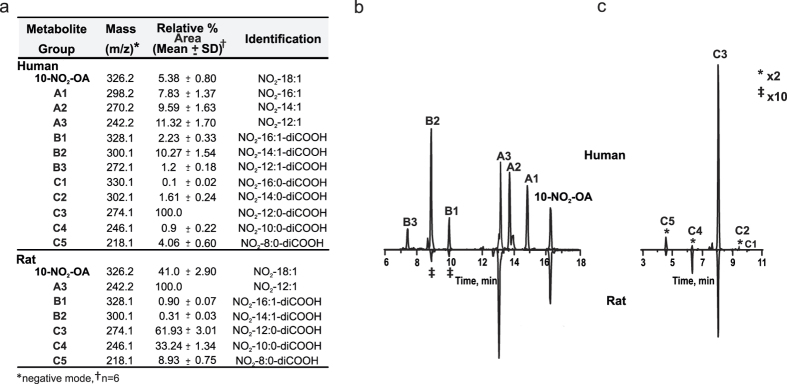
(**a**) Relative peak areas and mass determinations for 10-NO_2_-OA and its metabolites in the negative ion mode 0–6 h after 10-NO_2_-OA dose. Comparative chromatographic profiles of: (**b**) 10-NO_2_-OA and its β-oxidation metabolites (A1 to A3) and ω-carboxylation products (B1 to B3) in human (upper panel) and rat (lower panel) urine, and (**c**) Saturated 10-NO_2_-OA ω-carboxylated derivatives in human (upper panel) and rat (lower panel) urine.

This group of metabolites is characterized by three related species (A1 to A3) corresponding to β-oxidation products of 10-NO_2_-OA (Fig. 1a,b, and Table 1). A1 eluted at 14.81 min and displayed *m*/*z* 298.2020 (theoretical m/z 298.2024, −1.2 ppm), consistent with the loss of 28 amu from the parent 10-NO_2_-OA. Thus, A1 was identified as the product of a single round of β-oxidation, as previously reported in mouse plasma^30^. Metabolite A2 displayed a retention time of 13.62 min and *m*/*z* 270.1709 (theoretical m/z 270.1711, −0.66 ppm), corresponding to a two β-oxidation cycles product. A1 and A2 were almost absent in rat urine 6 h after the first gavage, but they became more intense 6 h after a second gavage. A3 had a retention time of 13.12 min and an experimental *m*/*z* of 242.1395 (theoretical 242.1398, −1 ppm) consistent with a molecular composition of C_12_H_20_NO_4_ corresponding to a three β-oxidation cycles derivative. A3 elicited the major mass spectrometric response in terms of area under the curve (AUC) for this series of metabolites in both rats and humans. Fragmentation analysis yielded no ions (carbon chain fragmentation) capable of providing additional structural data for this series of metabolites. The chromatographic peak obtained for the metabolite A2 in human urine presented an additional peak at 13.81 min (18% of total A-2) with identical molecular composition and neutral loss of 47 amu (HNO_2_) that was not further characterized (Fig. 1b). High resolution MS characterization for all metabolites in group A is presented in Sup Fig. 1.

### Di-carboxylated metabolite group B

**Figure 2:**
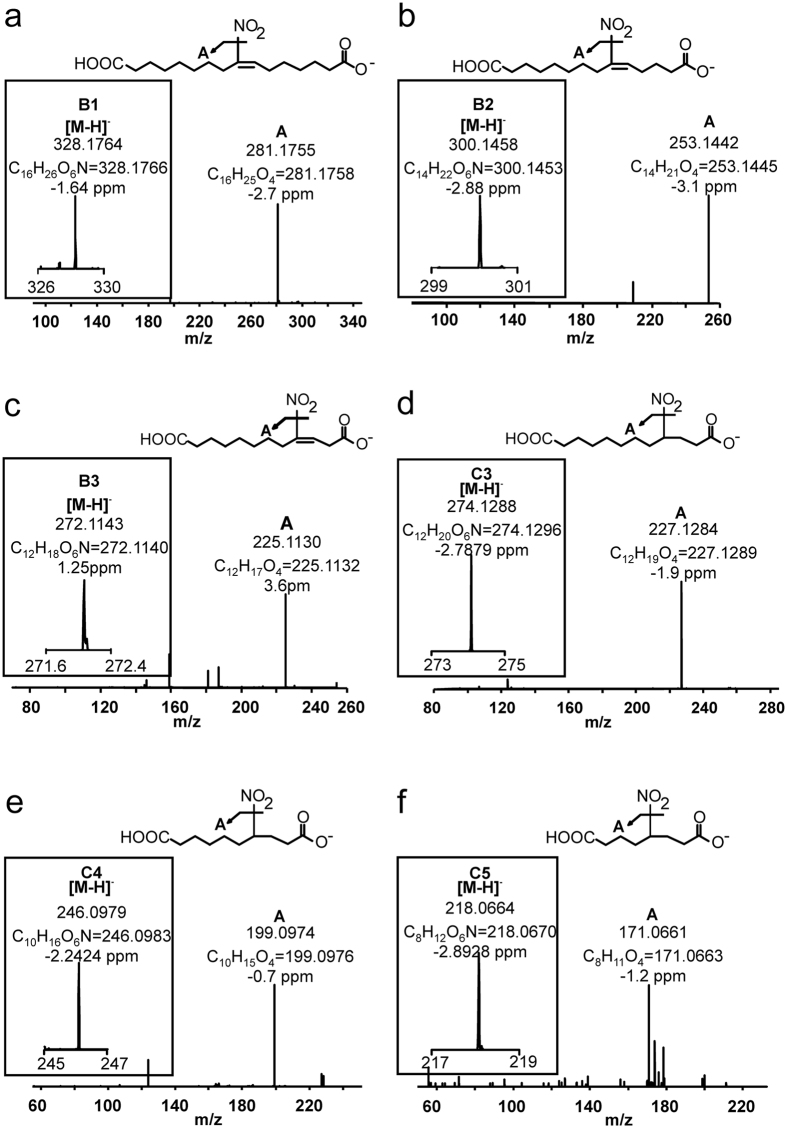
High resolution MS (insert) and fragmentation analyses for ω-carboxylated nitroalkene and nitroalkane species present in 0–6 h urine. (**a**) NO_2_-16:1-diCOOH, (**b**) NO_2_-14:1-diCOOH, (**c**) NO_2_-12:1-diCOOH, (**d**) NO_2_-12:0-diCOOH, (**e**) NO_2_-10:0-diCOOH and (**f**) NO_2_-8:0-diCOOH.

**Figure 3:**
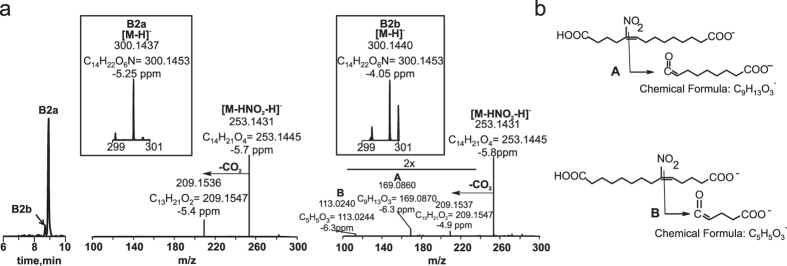
High resolution LC-MS/MS characterization of a ω-carboxylated 10-NO_2_-OA nitroalkene derivative with two rounds of β-oxidation present in 0–6 h urine. (**a**) Chromatographic profile, exact mass (insert) and fragmentation analysis for B2a and B2b. (**b**) Chemical structure of diagnostic nitroalkene fragmentation products for B2b.

This group includes ω–carboxylated species derived from both the parent 10-NO_2_-OA and metabolites from group A. Two metabolites were detected in rat urine collected during the first 6 h after administration with retention times of 10.45 min (B1) and 8.8 min (B2) (Fig. 1a,b and Table 1). B1 and B2 lost NO_2_^−^ ions upon fragmentation and had *m*/*z* values consistent with ω–end carboxylation of metabolites A-1 and A2 respectively. While the peak heights observed in the chromatograms for these metabolites were small in rats (0.1–0.2% of highest detected peak), they were more prominent in humans (2 and 7.9% respectively). The retention times for both rat- and human-derived B1 and B2 were identical, motivating a thorough characterization of these metabolites. B1 had a *m*/*z* of 328.1764 consistent with a composition of C_16_H_26_O_6_N^−^ (theoretical *m*/*z* 328.1766, −1.6 ppm), indicative of the presence of a double bond (Fig. 2a). Ion trap and triple quadrupole fragmentation resulted in the expected neutral HNO_2_ loss and charged loss of NO_2_^−^ respectively, which was further confirmed by the detection of an ion with *m*/*z* 281.1755 (theoretical 281.1758, −2.7 ppm) upon CID using HR-MS/MS. B2 presented a main peak at 8.8 min (B2a) with a shoulder at 8.6 min (B2b) (Figs 1b and 3). Both species had a *m*/*z* of 300.1458 (theoretical 300.1453, −2.9 ppm) and lost both NO_2_^−^ (detected using the triple quadrupole) and C_14_H_21_O_4_^−^ upon fragmentation. The later corresponded to a neutral loss of HNO_2_ (observed mass 253.1431, theoretical mass 253.1445 at −3.1 ppm) (Fig. 2b). In addition to HNO_2_, both B2a and B2b lost CO_2_ as confirmed at the 5 ppm level (Fig. 3a). Interestingly, whereas B2a did not yield any additional ions, fragmentation of B2b generated two structure-confirming products with *m*/*z* 169.0860 and 113.0240, consistent with an atomic composition of C_9_H_13_O_3_^−^ (theoretical 169.0870, −6.3 ppm) and C_5_H_5_O_3_^−^(theoretical 113.0244, −6.3 ppm). These ions correspond to carbon chain fragmentation products reflecting the occurrence of β-oxidation from either the ω- or α-ends, respectively (Fig. 3b). Further fragmentation of the *m*/*z* 253 ion obtained from both B2a and B2b MS/MS did not yield further structural information beyond additional CO_2_ and H_2_O losses.

Metabolite B3 corresponds to a species that underwent three rounds of β–oxidation in addition to ω-carboxylation. This metabolite, and displayed a *m*/*z* of 272.1143 (theoretical 272.1140, 1.2 ppm) corresponding to a molecular composition of C_12_H_18_O_6_N^−^ (Figs 1b and 2c). In Table 1 B3 is present in H-R fragmentation resulted in neutral and charged losses of NO_2_^−^ as confirmed by HR-MS and triple quadrupole detection. Carbon chain fragmentation produced low intensity ions thus precluding additional structural characterization.

### Nitroalkane metabolite DiCOOH group C

Metabolites in this group correspond to the most abundant group of metabolites (as evaluated by AUC) and presented the characteristic neutral loss of 47.0007 (HRMS-MS) and a NO_2_^−^ ion (*m*/*z* 46) upon fragmentation (Fig. 1a and c). Overall, metabolites in this series displayed *m*/*z* consistent with the reduction of the nitroalkene moiety to a nitroalkane and the presence of ω-end carboxylation. In rat-derived urine samples, 8, 10 and 12 carbon-long metabolites were identified while in humans additional metabolites with 14 and 16 carbons were also detected. C1 (NO_2_-16:0-diCOOH) and C2 (NO_2_-14:0-diCOOH) were only observed in human urine, displayed RT of 10.1 and 9.6 min respectively, and generated both the [HNO_2_] and the NO_2_^−^ characteristic losses upon collision-induced fragmentation Fig. 2d–f does not show C1 and C2 but C3-C4-C5. As previously observed, nitroalkane-containing molecules do not yield carbon chain fragmentations resulting in no additional structural information^33^. C3 corresponds to a metabolite that underwent three cycles of β-oxidation, plus ω-carboxylation and reduction of the nitroalkene moiety. This metabolite elicited the highest AUC responses in both human and rat samples. Metabolites C4 and C5 displayed RT of 6.3 and 4.5 min and had *m*/*z* consistent with four and five rounds of β-oxidation respectively. In contrast to non-quantitative AUC values, C5 was observed to be the most abundant metabolite by radiochromatographic assays.

### N-Acetylcysteine conjugates, metabolite group D

**Figure 4:**
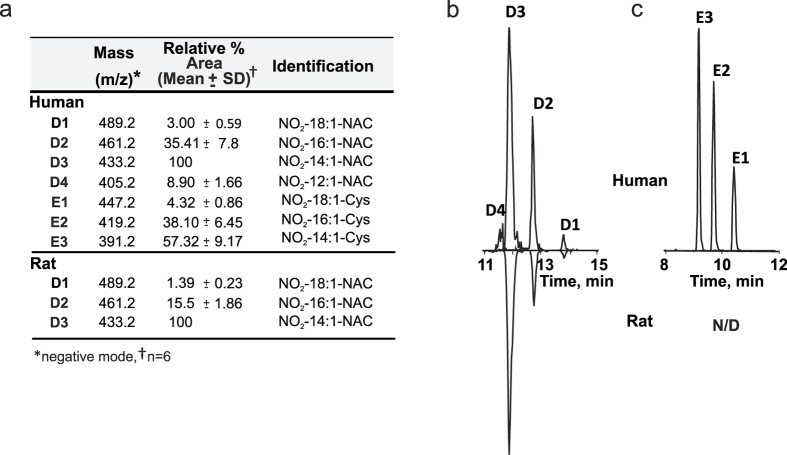
(**a**) Relative peak areas, and mass determinations for urinary 10-NO_2_-OA conjugates and its metabolites in the negative ion mode present in 0–6 h urine after 10-NO_2_-OA dose. Comparative chromatographic profiles of: (**b**) N-acetylcysteine and (**c**) cysteine conjugates of 10-NO_2_-OA and β-oxidation metabolites in human (upper panels) or rat (lower panels) urine.

This group includes N-acetylcysteine conjugates of 10-NO_2_-OA and nitroalkene-containing metabolites. Three metabolites, D1, D2 and D3 were identified in rat and human urine corresponding to N-acetylcysteine conjugates of 10-NO_2_-OA, A1 and A2, with the thioether bond located at carbons 9, 7 and 5, respectively (Fig. 4a,b). The location of the thioether bond was inferred based on the fact that 10-NO_2_-OA has only one electrophilic center located at C9. Fragmentation of these metabolites in the negative mode yielded an ion with *m*/*z* 162 corresponding to N-acetylcysteine in all three cases. This fragmentation pattern favoring the charged detection of N-acetylcysteine impaired additional structural analysis as it did not allow for further fatty acid backbone fragmentation. Finally, in human urine samples, an additional metabolite (D4) that corresponds to the N-acetylcysteine conjugates of metabolite A3 was identified with the thioether bond located on carbon 3.

### Cysteine conjugates, metabolite group E

Cysteine conjugates were only detected in human urine and were not present in the urine from rats. Among these metabolites, conjugates of 10-NO_2_-OA, A1 and A2 were detected with the thioether bond present at positions 9, 7 and 5 (E1, E2 and E3 respectively) (Fig. 4a,c). Consistent with previous reports, these metabolites exhibited charged loss of cysteine as detected by an *m*/*z* of 120.1^11^. E3 elicited the highest mass spectrometric response followed by E2 and E1. The retention times for these metabolites were significantly shorter than those observed for the corresponding D metabolites (~3 min) likely as a consequence of increased hydrophobicity resulting from the terminal amino group acetylation in D metabolites. As observed for D metabolites, preferential neutral loss of the lipid through β-elimination impaired to ability to further characterize these metabolites by MS/MS fragmentation.

### Taurine conjugates, metabolite group F

**Figure 5:**
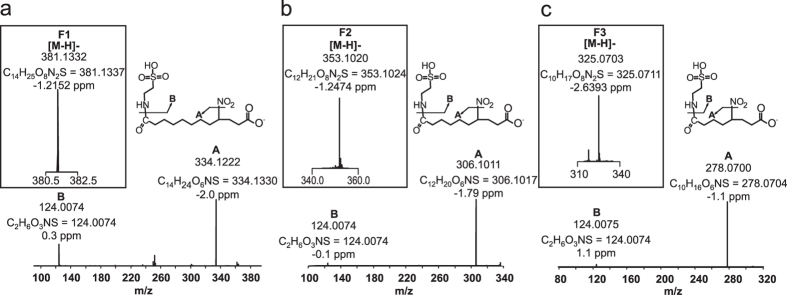
High resolution MS/MS analysis of taurine conjugates present in 0–6 h urine (**a**) NO_2_-12:0-diCOOH (**b**) NO_2_-10:0-diCOOH and (**c**) NO_2_-8:0-diCOOH.

Evaluation of metabolites derived from [^14^C] 10-NO_2_-OA administration to rats revealed a prominent ion characterized by odd *m*/*z* and the presence of a NO_2_ group that was absent in control urine. Further characterization allowed the identification of three metabolites F1, F2 and F3 with masses consistent with taurine conjugates of C3, C4 and C5 (Fig. 5a–c, respectively). Molecular composition analysis indicated the presence of 2 nitrogen, 1 sulfur and 8 oxygen atoms at the 2.6 ppm level. Fragmentation of F1 (C_14_H_25_O_8_N_2_S, *m*/*z* 381.1332) in negative ion mode resulted in a major neutral loss of HNO_2_ (47.0007) and a minor charged loss of 124 amu consistent with [C_2_H_6_O_3_NS]^−^, indicating the presence of a nitro group and taurine conjugation (Fig. 5a). Further fragmentation of the resulting ion did not provide additional structural information. Fragmentation of F2 and F3 rendered a similar fragmentation pattern with major neutral losses of HNO_2_ and minor charged losses of [taurine-H]^−^ (Fig. 5b and c, respectively).

### Sulfated monocarboxylates, metabolites group G

**Figure 6:**
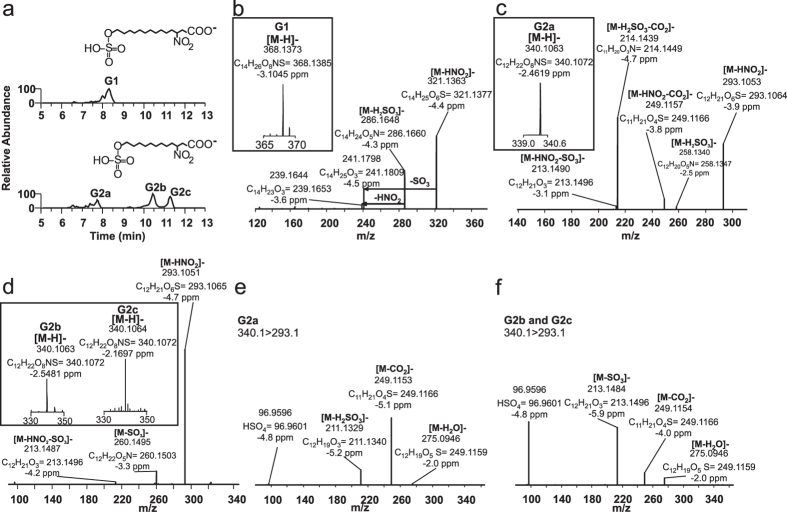
(**a**) Chromatographic profiles of 14- (upper panel) and 12 carbon-long chain (lower panel) sulfated nitro-fatty acid metabolites from rat 0–6 h urine. (**b**) High resolution MS (insert) and MS/MS analysis of G1, (**c**) G2a and (**d**) G2b-c metabolites. (**e**) High resolution MS^3^ analysis of G2a and (**f**) G2b-c daughter ions following HNO_2_ neutral loss.

Differential untargeted screening for 10-NO_2_-OA derivatives permitted the detection of four additional metabolites that were present in post-dose rat urine samples but not in pre-dose, controls or human samples. Two sets of peaks with *m*/*z* values 28.0310 amu apart were detected, suggesting the occurrence of one round of β-oxidation. Metabolite G1 displayed a mass of 368.1373 amu with a molecular composition of C_14_H_26_O_8_NS and an elution time of 8.3 min with a shoulder at 8.0 min (Fig. 6a, upper panel). CID fragmentation resulted in a major loss of 47.0010 amu (HNO_2_, theoretical mass 47.0007) and neutral losses of 81.9725 amu (H_2_SO_3_, theoretical mass 81.9725), 126.9575 amu (-HNO_2_-SO_3_, theoretical mass 126.9564) and 128.972 amu (-HNO_2_-H_2_SO_3_, theoretical mass 128.9720) (Fig. 6b). G2 was represented by three different isobaric peaks displaying RT of 7.6–7.7 min (G2a), 10.4 (G2b) and 11.4 min (G2c) (Fig. 6a, bottom panel). G2a had major neutral losses of 125.9624 amu (-H_2_SO_3_-CO_2_) and 47.0010 amu (HNO_2_) and minor losses of 90.9906 amu (-HNO_2_-CO_2_) and 81.9723 amu (-H_2_SO_3_). G2b and G2c presented an identical fragmentation pattern (Fig. 6d) characterized by a major loss of HNO_2_ (47.0007 amu) and minor losses of HNO_2_-SO_3_ (126.9576 amu) and SO_3_ (79.9568 amu). To further characterize these species, HR-MS^3^ spectra were obtained by fragmentation of daughter ions *m*/*z* 293.1, resulting from the neutral loss of HNO_2_. All G2 metabolites yielded the same MS^3^ fragments but with different intensities (Fig. 6e,f), with a major neutral loss of CO_2_ for G2a, and major losses of neutral SO_3_ and charged HSO_4_ for G2b/c.

### Sulfated monocarboxylates, metabolites group H

**Figure 7:**
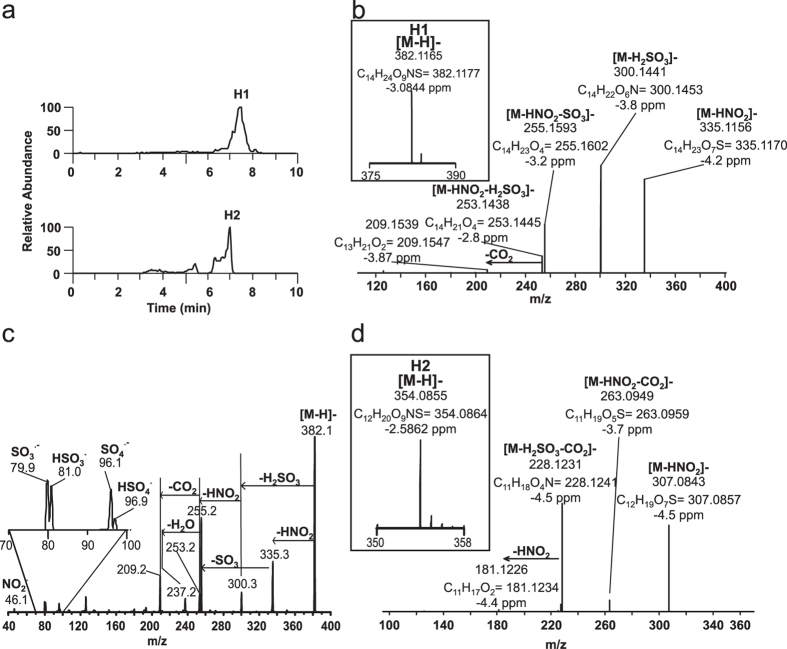
Sulfated 10-NO_2_-OA derivatives containing either keto or hydroxy groups from 0–6 h rat urine. (**a**) Chromatographic profiles for 14- (upper panel) and 12-carbon long (lower panel) metabolites. (**b**) High resolution MS (insert) and MS/MS spectral data and (**c**) Triple quadrupole MS/MS analysis for H1 in rat urine. (**d**) High resolution fragmentation analysis for urinary H2.

In addition to the G cluster, two metabolites identified as H1 and H2 were observed at slightly shorter RT than G1 and G2a (Fig. 7a). H1 displays an *m*/*z* 382.1165 consistent with a molecular composition of C_14_H_24_O_9_NS (theoretical 382.1177, −3 ppm). The shorter RT (7.4 min versus 8.3) and Δ mass of 13.9788 (theoretical 13.9792 with proposed compositions) versus G1 suggest the presence of either an additional carbonyl, or both a hydroxyl group and an unsaturation. Importantly, this Δ mass is not consistent with α-oxidation as the theoretical mass of the CH_2_ fragment is 14.0156 amu. Fragmentation analysis of H1 resulted in a similar pattern to that observed for G1, including neutral losses of HNO_2_, H_2_SO_3_, HNO_2_-SO_3_ and HNO_2_-H_2_SO_3_ (detected by HR-MSMS, Fig. 7b) and charged losses of NO_2_^−^, HSO_3_^−^ and HSO_4_^−^ (Fig. 7c). H2 displayed a Δ mass of 28.0306 (theoretical 28.0313, C_2_H_4_) with respect to H1, and losses of HNO_2_, HNO_2_-CO_2_, H_2_SO_3_-CO_2_ and H_2_SO_3_-CO_2_-HNO_2_ (Fig. 7d). The observation that H1 and H2 exhibit similar fragmentation patterns as G1 and G2, suggests that these are related species possibly containing an additional carbonyl moiety. Attempts to further define the position of the carbonyl group were unsuccessful as no carbon chain fragmentation was obtained for these molecules. The presence of a keto group usually induces carbon chain fragments in fatty acyl chains, although to a lesser extent than hydroxyl groups. As no evidence for this is observed, the presence of an aldehyde group is suggested.

### Odd-carbon number metabolites group I

**Figure 8:**
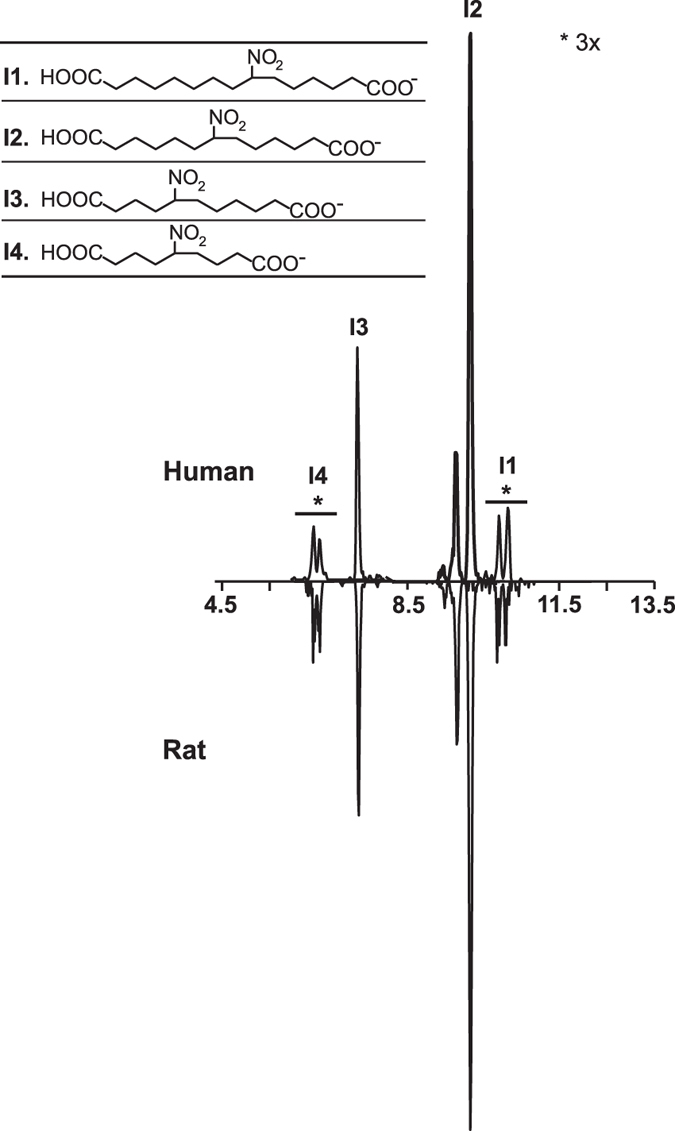
Chromatographic profiles of odd-carbon number metabolites found in both human and rat urine.

Targeted metabolite screening for charged losses of *m*/*z* 46 (NO_2_^−^) resulted in a series of metabolites present in both human- and rat-derived urine samples with RT of 10.84 and 11.09 min (I1, *m*/*z* 316.2), 9.87 and 10.21 min (I2, *m*/*z* 288.2), 7.60 min (I3, *m*/*z* 266.2) and 6.42 and 6.80 min (I4, *m*/*z* 232.1) (Fig. 8) corresponding to metabolites with odd number of carbons. I2 elicited the highest mass spectrometric response in both in rat and human, followed by I3, with small contributions of I1 and I4. HR-MS determinations of human I2 and I3 showed a mass of 288.1438 amu (determined for both peaks, theoretical 288.1453 amu, [C_13_H_22_O_6_N]^−^) and 260.1126 amu (determined for both peaks, theoretical 260.1140 amu, [C_11_H_18_O_6_N]^−^), respectively (data not shown). Elemental composition analysis suggests that these molecules are dicarboxylic nitroalkane metabolites. Ion trap fragmentation of I2 confirmed the neutral loss of HNO_2_ during CID. The peak intensities for I1 and 4 were too low for accurate mass determinations and composition evaluation. Rat metabolites exhibited the same retention times as human derivatives and were not further characterized.

### Determination of electrophilic metabolites in urine

**Figure 9:**
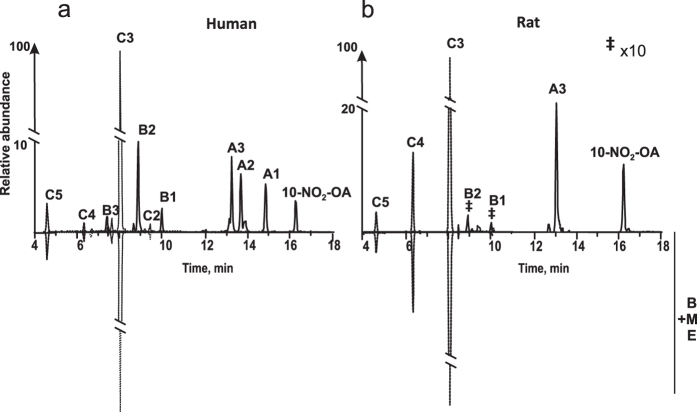
Representative HPLC-ESI-MS/MS chromatograms before and BME treatment of 10-NO_2_-OA metabolites in (**a**) human and (**b**) rat urine. Excess BME treatment leads to the complete consumption of electrophilic 10-NO_2_-OA derivatives as evidenced by the disappearance of precursor peaks from their original retention times (lower panels).

Whereas unsaturated nitroalkene-containing metabolites are expected to be electrophilic, their corresponding saturated nitroalkane derivatives are not. To determine the presence of electrophilic 10-NO_2_-OA derivatives in urine, metabolic profiling analysis was performed before and after beta-mercaptoethanol (BME) treatment (Fig. 9 upper and lower panel respectively). As expected, all metabolites from groups A and B disappeared after BME treatment, while metabolites in group C remained unaltered. Metabolites in other groups that did not contain unsaturations were not tested.

## Discussion

Electrophilic nitrated fatty acids (NO_2_-FA) are byproducts of gastric acidification and are formed acutely during inflammation^1^,^2^,^3^,^4^,^8^. From a functional perspective, NO_2_-FA induce Nrf2-dependent gene expression^19^,^34^,^35^, activate heat shock responses^19^, inhibit TLR4^18^ and NF-κB^17^ signaling as well as partially activate PPARγ^26^,^36^. As a result, administration of NO_2_-FA in animal models of disease results in tissue and organ protection during ischemia-reperfusion^10^,^21^, inflammation^18^,^22^,^37^,^38^, autoimmune activation and infection (*unpublished results*). Among this class of fatty acids, five Phase I clinical trials related to acute and chronic kidney injury were recently completed with 10-NO_2_-OA^20^. The unprecedented development of a free radical oxidation product into a promising human therapeutic agent is the motor for the exhaustive characterization of rat and human pathways for 10-NO_2_-OA bio-elimination presented herein. In this regard, although some aspects of 10-NO_2_-OA metabolism had previously been outlined in murine plasma^30^, a profound study that quantitatively and qualitatively evaluates the magnitude and pathways involved in 10-NO_2_-OA bio-elimination was lacking. Furthermore, the metabolism of 10-NO_2_-OA in the human population was completely unknown. Thus, the findings of this work not only shed light on the enzymes and pathways involved in 10-NO_2_-OA metabolism and inactivation, but also identify novel metabolites that might facilitate the future discovery of yet unidentified nitrated fatty acids.

Oral administration of radiolabeled [^14^C]-10-NO_2_-OA to rats revealed that an overall 35% of this molecule is excreted through urine. This confirms extensive absorption and rapid metabolism, as only traces of the native 10-NO_2_-OA molecule were detected in urine (not detected using radiochromatography) and 89% of total urine excretion occurred during the first 24 h. Beta-oxidation is a well-known metabolic route for fatty acids, fatty acid-derived signaling species (*e*.*g*. prostaglandins, leukotrienes, hydroxyl- and keto- derivatives), and also NO_2_-FA^39^. In fact, β-oxidation products of NO_2_-OA have been described in mouse plasma^30^. Beta oxidation can occur both in mitochondria and peroxisomes through the initial activity of very long chain acyl-CoA dehydrogenases or peroxisomal acyl-CoA oxidases respectively^39^. Metabolism is followed by the activity of a trifunctional enzyme in mitochondria (with hydratase, dehydrogenase and thiolase functions) or a bifunctional protein in peroxisomes that acts as a hydratase and dehydrogenase that is subsequently followed by a 3-ketoacyl-CoA thiolase. Of note, short chain fatty acid degradation (below 8 carbons) occurs exclusively in mitochondria with no peroxisomal participation^40^. Short acyl-CoA are exported and further processed in mitochondria. During the β-oxidation process, acyl-CoA conjugates are also substrates for acyl-CoA thioesterases^41^, that hydrolyze these conjugates to release CoA and a fatty acid anion. The absence of metabolites shorter than 8 carbons may indicate an impossibility of mitochondria to process short chain fatty acids containing a nitro group, attributed to steric hindrance caused by the vicinity of the nitro group to the carboxyl moiety. Inability to further process the 8 carbon nitro fatty acid chain is further supported by the low levels of expired radioactivity (the ^14^C label was located in C10), indicating that further oxidation is not favorable.

The analysis of metabolites includes structural characterization and high resolution mass spectrometry. Structural information regarding position of the NO_2_^−^ group can only be derived when collision induced dissociation generates carbon chain fragmentation. Following previously reported fragmentation analysis of nitrated fatty acids, these processes only result in informative fragments in the presence of a nitroalkene moiety which yields nitrile and carboxyl groups upon collision induced dissociation^33^. Unfortunately, reduced metabolites containing nitroalkane groups did not produce fragments that would allow further structural characterization. A similar situation was observed for Michael conjugates (cysteine and N-acetyl cysteine adducts) where fragmentation proceeded through neutral loss of the fatty acid portion of the molecule (β-elimination reaction).

Nitroalkene-containing fatty acids are electrophiles that modify thiols through Michael addition reactions^8^. Importantly, this is a reversible reaction with glutathione being the ultimate nitroalkene acceptor in an apparently enzyme-independent process, as glutathione S-transferases have been found not to be required^7^,^42^. Nitroalkene glutathione conjugates are exported from cells and into the circulation by multidrug resistance-associated proteins^42^, where hepatic γ-glutamyl transpeptidases and renal cysteinyl-glycine dipeptidase or aminopeptidase M generate the corresponding cysteine derivatives. No metabolites were found to be covalently bound to proteins in urine samples. In human urine, Cys-conjugates are the main metabolites of endogenously formed nitro-conjugated linoleic acid (NO_2_-CLA) with N-acetylcysteine conjugates also detected albeit at lower levels^11^. In contrast, while Cys-conjugates were prominent 10-NO_2_-OA metabolites in human urine, significant levels of the N-acetylcysteine derivatives were also present. Notably, only N-acetyl cysteine conjugates were detected as glutathione-derived metabolites in rat urine.

Reduction of the nitroalkene moiety to a nitroalkane is the main inactivation pathway for NO_2_-FA, and occurs during liver first pass in a reaction catalyzed by prostaglandin reductase-1^43^. Consistent with previous observations with endogenous NO_2_-CLA^11^, whereas free 10-NO_2_-OA and its β-oxidation products were detected both in human and rat urine, no saturated nitroalkane monocarboxylates were observed in these samples. Therefore, it is postulated that the presence of nitroalkene-containing fatty acids in urine derives from the excretion of N-acetylcys- and Cys-conjugates. Once in the bladder, these adducts reach new equilibria through thiol β-elimination with the consequential release of the nitroalkene fatty acid^11^. Thus, only adducted NO_2_-FA would be filtered by the kidneys while non-electrophilic nitroalkane fatty acids can be excreted through feces or renal active transport (e.g. dicarboxylates, taurine and sulfo cojugates). In addition to saturation, the liver also mediates nitroalkene esterification into complex lipids which are then packaged into lipoproteins particles and distributed in the circulation^31^. No complex lipid metabolites of NO_2_-OA were detected in either human or rat urine, as this is not a major route of excretion for these species. In this regard, initial rat fecal analysis indicated the major presence of metabolite A1 and additional hydrophobic metabolites (data not shown). These metabolites most likely stem from biliary excretion, but a microbiome component in the metabolism of fecal samples cannot be discarded without further investigation.

Formation of dicarboxylic derivatives is a common route for fatty acid metabolism; however, its involvement in nitro-fatty acid bio-elimination was not previously recognized. Following an initial ω-hydroxylation step typically catalyzed by the CYP4A family of cytochrome P450-dependent enzymes and its oxidation to a dicarboxylic acid in the cytosol, fatty acids are catabolized by sequential rounds of β-oxidation at both the α- and ω-ends of the molecule^40^. In humans, dicarboxylic-CoA’s are exclusively oxidized in peroxisomes^44^,^45^,^46^, while in rats, an additional participation of pristanoyl-CoA oxidase has been reported^45^. Quantitative radiochromatographic analysis identified 4-nitro-octadienoic acid (C5) as the major metabolite found in urine accounting for 7.81% of the total radiolabeled 10-NO_2_-OA administered. Nonetheless, this metabolite only elicited a small response in the mass spectrometer, with the four carbon longer C3 metabolite generating the highest signal. This is a common issue in LC-MS/MS analysis and is particularly prevalent in urine samples, where the detection of the more polar metabolites is impaired by the presence of charged solutes that result in ionization dampening at the MS source. A series of odd-carbon number dicarboxylic nitroalkane metabolites were also identified in human and rat urine. The origin of these species is unknown but their presence might suggest that nitrated fatty acids could be metabolized by peroxisomal α-oxidation^47^.

The detection of nitro-fatty acid taurine adducts in urine was unexpected, as taurine conjugates are thought to be preferentially disposed through the bile. Notably, nitro-fatty acid taurine conjugates are present in rat urine but were undetectable in human samples. Using rat hepatocytes, it was previously shown that prostaglandin E1, E2 and its β-oxidation metabolites (dinor-PGE1 and dinor-PGA2) are modified to taurine conjugates^48^. However, whether nitro-fatty acid taurine adducts are formed in humans or if they are exclusively excreted through bile, is currently unclear. Of interest, considering that taurine is an abundant bioactive amino acid in brain tissue and acyl taurine conjugates have been described, this finding may have implications in central nervous system in rats but not in humans^49^. Finally, the presence of glucuronates and glycine adducts was not apparent in either rat or human urine as determined by targeted and untargeted LC-MS/MS analysis.

Nitrated fatty acids are formed as a consequence of ^•^NO_2_ production secondary to peroxynitrite or dinitrogen trioxide homolysis, NO_2_^−^ reaction with peroxidase ferryl heme centers and by nitric oxide autoxidation^1^,^3^,^6^. Free radical generation and the occurrence of imbalances between oxidant production and antioxidant defenses (*i*.*e*. oxidative stress) are traditionally viewed as a deleterious processes causatively associated with the development of acute and chronic pathological conditions^50^. However, in the past years our knowledge of biological redox processes has expanded greatly and it is now evident that controlled oxidant generation is not only an important mechanism for environmental adaptation but also that it has essential roles in many aspects of cellular communication^51^. In this regard, the development of 10-NO_2_-OA as a candidate therapeutic agent for clinical use constitutes a significant milestone in the continuing evolution of this paradigm, and reinforces the notion that the generation of potent oxidizing species can generate molecules that display beneficial pro-homeostatic functions in biological systems.

## Additional Information

**How to cite this article**: Salvatore, S. R. *et al*. Evaluation of 10-Nitro Oleic Acid Bio-Elimination in Rats and Humans. *Sci. Rep.* **7**, 39900; doi: 10.1038/srep39900 (2017).

**Publisher's note:** Springer Nature remains neutral with regard to jurisdictional claims in published maps and institutional affiliations.

## Supplementary Material

Supplementary Data

## Figures and Tables

**Table 1:** Mass, retention time, chemical composition and analysis description for all identified metabolites in human (H) and rat urine (R).

	Metabolite Group	Mass (m/z)*	Retention Time (min)	Composition*	Analysis MRM HR-MS HR-MS^2^			Urine	Identification
A	10-NO_2_-OA	326.2	16.22	C_18_H_32_O_4_N	✓	✓	✓	H-R	NO_2_-18:1
	1	298.2	14.81	C_16_H_28_O_4_N	✓	✓	✓	H	NO_2_-16:1
	2	270.2	13.62	C_14_H_24_O_4_N	✓	✓	✓	H	NO_2_-14:1
	3	242.2	13.12	C_12_H_20_O_4_N	✓	✓	✓	H-R	NO_2_-12:1
B	1	328.1	10.45	C_16_H_26_O_6_N	✓	✓	✓	H-R	NO_2_-16:1-diCOOH
	2	300.1	8.80	C_14_H_22_O_6_N	✓	✓	✓	H-R	NO_2_-14:1-diCOOH
	3	272.1	7.40	C_12_H_18_O_6_N	✓	✓	✓	H	NO_2_-12:1-diCOOH
C	1	330.1	10.7	C_16_H_28_O_6_N	✓	✓		H	NO_2_-16:0-diCOOH
	2	302.1	9.63	C_14_H_24_O_6_N	✓	✓		H	NO_2_-14:0-diCOOH
	3	274.1	8.11	C_12_H_20_O_6_N	✓	✓	✓	H-R	NO_2_-12:0-diCOOH
	4	246.1	6.30	C_10_H_16_O_6_N	✓	✓	✓	H-R	NO_2_-10:0-diCOOH
	5	218.1	4.50	C_8_H_12_O_6_N	✓	✓	✓	H-R	NO_2_-8:0-diCOOH
D	1	489.2	13.68	C_23_H_41_O_7_N_2_S	✓			H-R	NO_2_-18:1-NAC
	2	461.2	12.60	C_21_H_37_O_7_N_2_S	✓			H-R	NO_2_-16:1-NAC
	3	433.2	11.80	C_19_H_33_O_7_N_2_S	✓			H-R	NO_2_-14:1-NAC
	4	405.2	11.48	C_17_H_29_O_7_N_2_S	✓			H	NO_2_-12:1-NAC
E	1	447.2	10.34	C_21_H_39_O_6_N_2_S	✓			H	NO_2_-18:1-Cys
	2	419.2	9.60	C_19_H_35_O_6_N_2_S	✓			H	NO_2_-16:1-Cys
	3	391.2	9.05	C_17_H_31_O_6_N_2_S	✓			H	NO_2_-14:1-Cys
F	1	381.1	7.43	C_14_H_25_O_8_N_2_S	✓	✓	✓	R	NO_2_-12:0-diCOOH-tau
	2	353.1	5.46	C_12_H_21_O_8_N_2_S	✓	✓	✓	R	NO_2_-10:0-diCOOH-tau
	3	325.1	3.09	C_10_H_17_O_8_N_2_S	✓	✓	✓	R	NO_2_-8:0-diCOOH-tau
G	1	368.1	8.30	C_14_H_26_O_8_NS		✓	✓	R	NO_2_-14:0-sulfo
	2a	340.1	7.70	C_12_H_22_O_8_NS		✓	✓	R	NO_2_-12:0-sulfo
	2b	340.1	10.50/11.20	C_12_H_22_O_8_NS		✓	✓	R	NO_2_-12:0-sulfo
H	1	382.1	7.50	C_14_H_24_O_9_NS		✓	✓	R	NO_2_-14:1-OH-sulfo or NO_2_-14:0-oxo-sulfo
	2	354.1	7.00	C_12_H_20_O_9_NS		✓	✓	R	NO_2_-12:1-OH-sulfo or NO_2_-12:0-oxo-sulfo
I	1	316.1	10.48	C_15_H_26_O_6_N	✓			H-R	NO_2_-15:0-diCOOH
	2	288.1	10.19	C_13_H_22_O_6_N	✓	✓		H-R	NO_2_-13:0-diCOOH
	3	260.1	7.48	C_11_H_18_O_6_N	✓	✓	✓	H-R	NO_2_-11:0-diCOOH
	4	232.1	6.42	C_9_H_14_O_6_N	✓			H-R	NO_2_-9:0-diCOOH

*Negative ion mode.
