# Supplementary material for: Evaluation of 10-Nitro Oleic Acid Bio-Elimination in Rats and Humans
Source: Sci Rep. 2017 Jan 5;7:39900. doi: 10.1038/srep39900 (PMC5215368; doi:10.1038/srep39900)
Supplement: Supplementary Data [file srep39900-s1.pdf]

Supplementary Material

EVALUATION OF 10-NITRO OLEIC ACID BIO-ELIMINATION IN RATS AND HUMANS

Sonia R. Salvatore[1], Dario A. Vitturi[1], Marco Fazzari[1,2], Diane K. Jorkasky[3] and Francisco J. Schopfer[1]

[1]Department of Pharmacology and Chemical Biology, University of Pittsburgh, Pittsburgh, PA 15261, USA. [2]Fondazione Ri.MED, Palermo, 90133, Italy. [3]Complexa Inc., Pittsburgh, PA, 15203, USA.

***Running title: Nitro-Fatty Acid Urinary Metabolites**

To whom correspondence may be addressed: Francisco J. Schopfer, Ph.D., Department of Pharmacology & Chemical Biology, Thomas E. Starzl Biomedical Science Tower E1343, 200 Lothrop St, University of Pittsburgh, Pittsburgh, PA 15213, USA; Tel: (412) 648-0193; Fax: (412) 648-2229; Email: fjs2@pitt.edu.

a

b

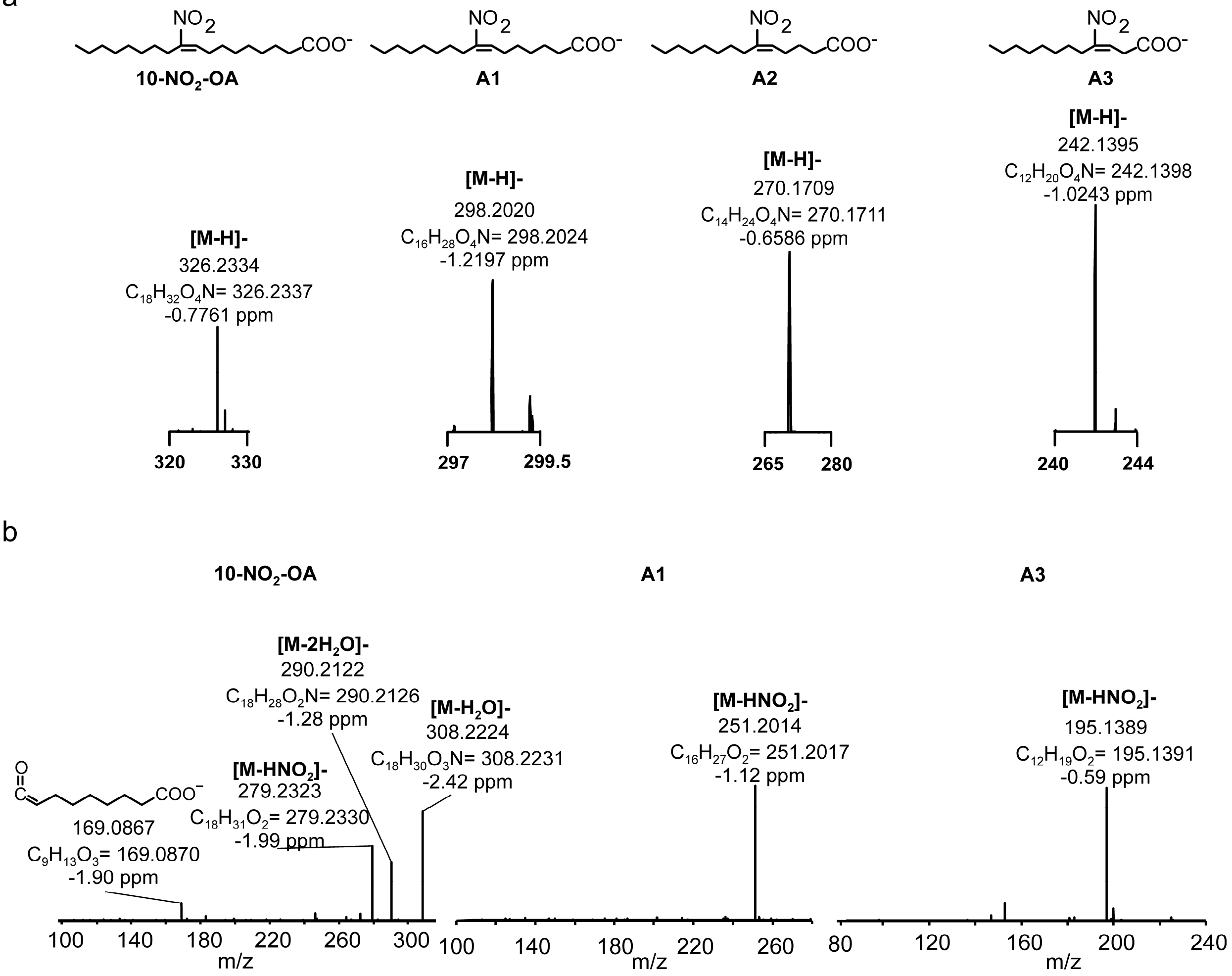

Supplementary Fig 1 a) Structures and corresponding high resolution MS analysis of 10-NO_2-OA and its β-oxidation metabolites (A1 to A3), **b)** MS/MS analysis for 10-NO_2-OA, A1 and A3 metabolites.
